# Association between DASH diet and metabolic syndrome in US adults: a cross-sectional study

**DOI:** 10.3389/fpubh.2025.1524399

**Published:** 2025-06-24

**Authors:** Jianing Liu, Rui Yang, Pei Ma, Xiaobo Zhu

**Affiliations:** ^1^The First Clinical College, Shandong University, Jinan, China; ^2^Department of General Pediatrics and Neonatology, The Second Hospital of Shandong University, Jinan, China; ^3^The Second Clinical Medical College, Cheeloo College of Medicine, Shandong University, Jinan, China; ^4^Children’s Medical Center, The Second Hospital of Shandong University, Jinan, China

**Keywords:** NHANES, metabolic syndrome, DASH diet, hypertension, insulin resistance

## Abstract

**Objective:**

Metabolic syndrome (MetS) is a global issue increasingly threatening human health and the quality of life. This study aimed to explore the relationship between the Dietary Approaches to Stop Hypertension (DASH) diet and MetS, with the goal of providing insights into how dietary interventions may be associated with MetS.

**Methods:**

We utilized data from the National Health and Nutrition Examination Survey 2005–2008 and divided DASH scores into five quintile groups. We included individuals over the age of 18 who underwent two 24-h dietary recalls administered by trained interviewers to calculate the DASH score. The chi-square test or Kruskal–Wallis test was used to compare the variable differences between the groups. A logistic regression model was used to analyze the association between DASH and MetS. The subgroup analysis was performed to explore the association between DASH diet and MetS in different populations. And we further explored the relationship between DASH and 5 particular MetS items in depth.

**Results:**

The final sample size was 8,780 individuals, with DASH scores ranging from 18 to 37. The proportion of women, younger individuals, non-Hispanic Blacks, low-income individuals, smokers, and those with MetS gradually decreased from Q1 to Q5 quintile groups. After adjusting for all variables, the odds ratio of MetS risk in the highest DASH quintile (Q5) compared with the lowest (Q1) was 0.60 (0.48, 0.77). The subgroup analyses revealed that the inverse association between DASH score and MetS risk was more pronounced among women, Whites, high-income individuals, those with a college degree or higher, those who were physically active, and never smokers. Subsequently, we discovered that DASH levels are significantly negatively correlated with waist circumference (WC), fasting plasma glucose (FPG), triglycerides (TG), and systolic blood pressure (SBP), while exhibiting a positive correlation with high-density lipoprotein cholesterol (HDL-C) through RCS curves and linear regression.

**Conclusion:**

Our findings imply that higher adherence to the DASH diet is inversely associated with the risk of MetS, with pronounced effects on WC, FPG, SBP, DBP, TG, and HDL.

## 1 Introduction

Metabolic syndrome (MetS) is a cluster of cardiovascular disease and diabetes-related risk factors, including hypertension, impaired fasting glucose, abdominal obesity, and dyslipidemia (1). MetS is a global issue increasingly threatening human health and the quality of life. Its incidence increases with age (2). Globally, the prevalence of MetS is approximately 25% and is increasing annually due to the growing trends of population aging and overweight/obesity (3). Studies have found that these risk factors manifest in childhood and adolescence, and are associated with a higher likelihood of chronic diseases in adulthood (4). Therefore, the gradual trend toward younger onset, the high prevalence of MetS, and its association with the risk of other chronic diseases, all indicate the clinical severity of MetS (5). Identification, diagnosis, and early intervention in MetS are topics of growing concern. MetS poses a significant public health challenge globally (6).

The etiology of MetS is not fully elucidated, but current research considers it as the result of the interplay between multiple genetic and environmental factors (7). This condition is influenced by various environmental factors, particularly a diet high in fats and carbohydrates, which can increase the occurrence of insulin resistance (8). In addition, low physical activity and a sedentary lifestyle also contribute to the development and progression of MetS (9). Furthermore, overweight or obesity is a major underlying cause of MetS, as these conditions are related to insulin resistance (2). Therefore, losing weight, reducing central obesity, reducing sedentary time, and developing a healthy diet are essential interventions that effectively help reduce the occurrence of metabolic abnormalities (10).

The Dietary Approaches to Stop Hypertension (DASH) diet is a dietary pattern aimed at lowering blood pressure (BP). Developed by Fung et al., the DASH diet promotes the consumption of vegetables, fruits, low-fat dairy, whole grains, nuts, fish, and poultry, while limiting the intake of red meat, sweets, and sugar-sweetened beverages (11). In addition, the DASH diet emphasizes reducing sodium intake and increasing the intake of potassium, magnesium, and calcium (11).

The DASH diet has a potential association with MetS (12). Studies have shown that adherence to the DASH diet can significantly reduce the risk of developing MetS (13, 14). The potential mechanisms by which the DASH diet may prevent MetS are likely related to its effects on insulin resistance, which is a key component of MetS and can lead to impaired glucose tolerance or non-insulin-dependent diabetes mellitus (15). The DASH diet provides abundant fiber, potassium, calcium, and magnesium, and limits the intake of total fat, saturated fatty acids, cholesterol, and sodium, thus helping improve insulin sensitivity and regulating blood glucose levels (16). In addition, the high intake of fruits and vegetables in the DASH diet provides a wealth of antioxidants, which are also crucial for correcting glucose and insulin abnormalities (17).

At present a shift in dietary patterns for preventing chronic diseases is gaining momentum (18). Studies on the relationship between dietary structure and metabolic diseases are gradually emerging (19, 20). However, there is relatively little research on the relationship between the DASH dietary pattern and metabolic syndrome. Therefore, in this study we use the National Health and Nutrition Examination Survey (NHANES) database from 2005 to 2018 to conduct an association analysis between the DASH diet and MetS, aiming to provide insights into how dietary interventions may be associated with MetS. Additionally, the study also pays attention to differences among various subgroups.

## 2 Materials and methods

### 2.1 Data sources

The NHANES database is a cross-sectional survey in the United States that uses a complex, multistage probability sampling design. The National Center for Health Statistics Research Ethics Review Board approved the NHANES protocol. Each participant signed a consent form. NHANES is updated every 2 years. We used publicly available data from 2005 to 2018 without personal identifiable information for this analysis. Interview questionnaires and examination data are publicly available. Participants were first interviewed in their homes, during which demographic information and a variety of health-related information were collected. One to 2 weeks later, subjects underwent a standardized physical examination, as well as additional investigations like exercise testing, 24-h dietary recall, and a blood draw in a mobile examination center. Blood samples were taken with the participant fasting. Participants who visited the examination in the morning were requested to fast for 9 h, those visiting in the afternoon or evening were requested to fast for 6 h.

### 2.2 Inclusion and exclusion criteria

**Figure 1:**
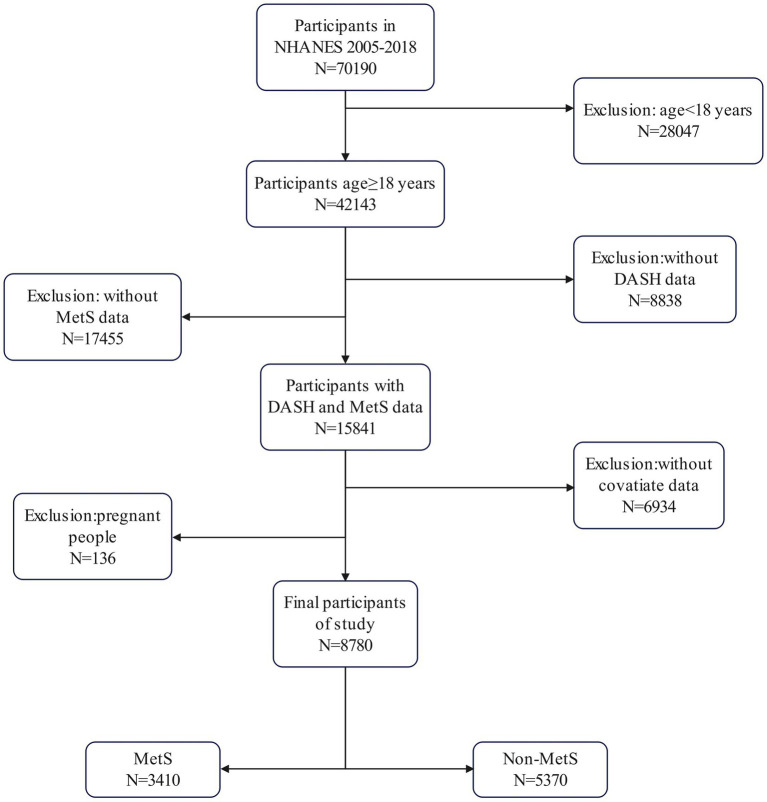
Flowchart of sample selection from the National Health and Nutrition Examination Survey (NHANES) 2005–2018.

The original total sample size was 70,190 and the age of the sample ranged from 0 to 85 years. We excluded 28,047 participants who were under 18 years of age, 8,838 participants without DASH data (The participants who only had one recall of 24-dietary intake were also excluded), 17,455 participants without MetS data (Any missing of the five components is excluded), and 6,934 participants without covariates (including education level, family poverty–income ratio (PIR), smoking status, alcohol consumption, age, ethnicity, and physical activity). Also, we excluded 136 pregnant people. This left a total of 8,780 participants in our cohort for analysis. The sample selection process is illustrated in Figure 1.

### 2.3 Data collection and preprocessing

(1) The demographic data, including sex, age, race, education level, and household poverty–income ratio (PIR), were collected. Participants with poverty–income ratio (PIR) < 1.3 were defined as living in poverty (21). (2) Measurements and laboratory data, comprising waist circumference (WC), systolic blood pressure (SBP), diastolic blood pressure (DBP), triglycerides (TG), high-density lipoprotein cholesterol (HDL-C), and fasting plasma glucose (FPG), were obtained. (3) Questionnaire data, including family history of diabetes, insulin use, antihypertensive medication use, lipid-lowering medication use, and history of coronary heart disease, cancer, myocardial infarction, and other major diseases, as well as physical activity indicators, were collected. Based on World Health Organization guidelines (22), participants engaged in more than 149 min of moderate physical activity, more than 74 min of vigorous physical activity, or more than 599 metabolic equivalent (MET) minutes per week were classified as active, with all others classified as inactive.

Dietary intake was assessed using two 24-h dietary recalls conducted by trained interviewers. All of the 8,780 subjects participated in the interview and dietary data analysis. The DASH score was based on the consumption of eight DASH food groupsor items: whole grains, vegetables, fruits, dairy, red meat, nuts/seeds/vegetables, sweets, and sodium. In terms of data collection, the first dietary recall was collected through a face-to-face interview at the Mobile Examination Center (MEC). The second interview was conducted by telephone 3–10 days after the first interview. During the interviews at the MEC, interviewers used a number of standard measuring tools, such as shape charts, rectangular grids, cups, bowls, spoons, rulers, etc., to help respondents estimate portion sizes of foods. However, these tools were not intended for specific foods (23). In addition, the R package “Dietaryindex” goes beyond a pure calculation tool - it provides a comprehensive and user-friendly environment for researchers by providing internal access to multiple data sets, including NHANES data (2005–2018), DASH study data. Participants were ranked according to their intake of each food group/food item and classified into quintiles. For whole grains, vegetables, fruits, dairy, and nuts/seeds/legumes, individuals in the 1st quintile were assigned a score of 1 and those in the 5th quintile were assigned a score of 5. For red meat, sweets, and sodium, the scores were reversed, with individuals in the 1st quintile assigned a score of 5 and those in the 5th quintile assigned a score of 1. The scores for each food group/food item were then summed to obtain an overall DASH fidelity score, ranging from 8 (least fidelity) to 40 (most fidelity) (24).

### 2.4 Definition of MetS

MetS was defined using the National Cholesterol Education Program-Adult Treatment Panel III (NCEP-ATPIII) (25) criteria, according to which the disease is present when 3 or more of the following risk factors are present: (1) increased WC, where WC is >88 cm in women and >102 cm in men; (2) high triglycerides, where triglycerides are >150 mg/dL (1.7 mmol/L); (3) low HDL-C, with HDL-C < 50 mg/dL in women and <40 mg/dL in men; (4) elevated blood pressure, with blood pressure >130/85 mmHg; and (5) elevated FPG, with FPG > 110 mg/dL or drug treatment for elevated FPG and previously diagnosed type 2 diabetes.

### 2.5 Statistical analysis

Given the complex, multi-stage, stratified sampling design of the NHANES database, we conducted a weighted analysis in accordance with the recommended weights of the database. The analysis was weighted, taking into account the intricate survey design, with weighting factors including the two-day dietary interview weight (WTDR2D), as well as stratification (SDMVSTRA) and primary sampling units (SDMVPSU). Additionally, we employed a missing data deletion method, accurately documenting the frequency of deleted missing variables.

In this study, mean ± standard deviation (SD) was used to summarize the continuous variables, while frequency (percentage, %) was used to describe the categorical variables. WC, SBP, DBP, TG, HDL, and FPG were expressed as mean ± SD, while age was expressed as median (IQR). To assess statistical differences, Student’s *t*-test or Kruskal-Wallis rank sum test was used. The categorical variables were described by the number of samples (P0-P25, P25-P50, P50-P75, and P75-P100).

Odds ratios and 95% confidence intervals (CIs) were calculated using multivariable logistic regression analysis to assess associations between DASH and metabolic syndrome. Three statistical models were used for this assessment. The crude model was not adjusted for any covariate, while in model 1 adjustments were made for age and sex. In Model 2, additional adjustments were made for race, poverty income rate (PIR), education level, diabetes status, physical activity, and smoking status, and trend tests were performed to further confirm the reliability of the results. Based on the fully adjusted model, subgroup analysis was performed to analyze the association between DASH score and MetS in different strata of age (18–50 years; 50–85 years), sex (male; female), physical activity (active physical activity; inactive physical activity), alcohol consumption (never drinking; ever drinking; mild drinking; moderate drinking; severe drinking), smoking status (never smoke; ever smoke; currently smoke), race (white; black; hispanic; other races), education level (some collage or AA degree; high school grad/GDE or equivalent;9-11th grade; less than 9th grade) and family PIR (middle income; low income; high income). A linear regression model was used to examine the association between DASH level and the values of six biochemical MetS indicators. β-coefficients and 95% confidence intervals (CIs) were calculated to assess the associations between DASH and WC, HDL, TG, FPG, SBP, and DBP. The significance of the interactions was assessed using the *p*-values for the interaction terms of the stratified factors. The nonlinear relationship between DASH and MetS and its components was tested using restricted cubic spline regression (RCS). The likelihood ratio test was used to confirm this relationship. All analyses were performed using R 4.4.1 (http://www.R-Project.org, R Foundation) and the freely available statistical version 1.3. Two-sided *p* values <0.05 were considered statistically significant.

## 3 Results

### 3.1 Baseline characteristics of the participants

The study sample consisted of 8,780 participants with DASH scores between 18 and 37. The DASH scores were divided into five quintiles: Quintile1 represented the lowest DASH scores between 18 and 24; Quintile2 represented low DASH scores between 24 and 25.5; Quintile3 represented medium DASH scores between 25.5 and 27; Quintile4 represented high DASH scores between 27 and 29; and Quintile5 represented the highest DASH scores between 29 and 37.

Table 1 shows the distribution of participant characteristics across the quintiles of DASH scores. The characteristics, including sex, race, poverty income ratio (PIR), education level, physical activity, smoking status, age, WC, systolic blood pressure (SBP), diastolic blood pressure (DBP), triglycerides (TG), high-density lipoprotein (HDL), showed significant differences among DASH quintile groups. The proportion of women progressively decreased (*p* < 0.001) from the lowest (56.9%) to the highest DASH score quintile (39.5%), while the proportion of men increased (from 43.1 to 60.5%). The proportion of white and black participants showed a decreasing trend across quintiles, while the proportion of Hispanic and other participants increased. The proportion of individuals with a college degree or higher education level gradually increased from the lowest (36.4%) to the highest DASH score group (46.8%), while the proportion of participants with other education levels decreased, but the proportion of individuals with less than a 9th grade degree did not. The proportion of participants with low income, current smokers, those with inactive physical activity, and those with MetS decreased from the lowest to the highest DASH score group. In addition, participants in the highest quintile of DASH score Q5 tended to have lower DBP, TG, and FPG levels and a smaller WC compared to the other quintiles. These results indicate that a higher DASH score is associated with more favorable trends in numerous health-related characteristics in the population.

**Table 1:** Baseline characteristics of the participants.

Variables	Level	Quintile1	Quintile2	Quintile3	Quintile4	Quintile5	*p*
Age (median [IQR])		44.00 [31.00, 59.00]	48.00 [34.00, 63.00]	52.00 [35.00, 65.00]	54.00 [39.00, 68.00]	60.00 [44.00, 70.00]	<0.001
Sex (%)	Male	909 (43.1)	858 (48.2)	877 (51.3)	979 (55.3)	856 (60.5)	<0.001
	Female	1,199 (56.9)	922 (51.8)	831 (48.7)	790 (44.7)	559 (39.5)	
Race (%)	White	1,009 (47.9)	789 (44.3)	755 (44.2)	780 (44.1)	605 (42.8)	<0.001
	Black	585 (27.8)	455 (25.6)	336 (19.7)	300 (17.0)	202 (14.3)	
	Hispanic	382 (18.1)	430 (24.2)	516 (30.2)	579 (32.7)	517 (36.5)	
	Other	132 (6.3)	106 (6.0)	101 (5.9)	110 (6.2)	91 (6.4)	
Family PIR (%)	Middle income	912 (43.3)	771 (43.3)	715 (41.9)	761 (43.0)	582 (41.1)	<0.001
	Low income	859 (40.7)	653 (36.7)	601 (35.2)	583 (33.0)	431 (30.5)	
	High income	337 (16.0)	356 (20.0)	392 (23.0)	425 (24.0)	402 (28.4)	
Education level (%)	Some college or AA degree	768 (36.4)	667 (37.5)	674 (39.5)	755 (42.7)	662 (46.8)	<0.001
	High school Grad/GED or equivalent	702 (33.3)	588 (33.0)	528 (30.9)	503 (28.4)	358 (25.3)	
	9–11th grade	472 (22.4)	339 (19.0)	302 (17.7)	286 (16.2)	191 (13.5)	
	Less than 9th grade	166 (7.9)	186 (10.4)	204 (11.9)	225 (12.7)	204 (14.4)	
Physical activity (%)	Inactive	1,585 (75.2)	1,306 (73.4)	1,205 (70.6)	1,226 (69.3)	945 (66.8)	<0.001
	Active	523 (24.8)	474 (26.6)	503 (29.4)	543 (30.7)	470 (33.2)	
Smoke status (%)	Never	896 (42.5)	806 (45.3)	878 (51.4)	960 (54.3)	859 (60.7)	<0.001
	Ever	471 (22.3)	459 (25.8)	467 (27.3)	496 (28.0)	429 (30.3)	
	Current	741 (35.2)	515 (28.9)	363 (21.3)	313 (17.7)	127 (9.0)	
Diabetes (%)	No	1,714 (81.3)	1,401 (78.7)	1,334 (78.1)	1,387 (78.4)	1,112 (78.6)	0.086
	Yes	394 (18.7)	379 (21.3)	374 (21.9)	382 (21.6)	303 (21.4)	
Waist circumference (cm) [mean (SD)]		102.38 (17.73)	101.80 (17.48)	101.31 (15.92)	99.81 (15.30)	98.13 (14.45)	<0.001
SBP (mmHg) [mean (SD)]		123.64 (17.85)	124.47 (18.41)	124.51 (18.62)	125.28 (18.38)	126.15 (19.56)	0.001
DBP (mmHg) [mean (SD)]		70.44 (12.05)	69.57 (11.97)	69.55 (11.47)	69.44 (12.09)	68.60 (11.45)	<0.001
TG (mg/dL) [mean (SD)]		132.44 (119.10)	131.65 (106.67)	133.79 (109.61)	134.85 (157.23)	124.95 (90.57)	0.178
HDL (mg/dL) [mean (SD)]		50.53 (15.32)	52.36 (15.32)	52.54 (15.40)	53.88 (15.61)	56.19 (16.17)	<0.001
FPG (mg/dL) [mean (SD)]		110.29 (37.72)	111.39 (38.22)	111.81 (37.34)	111.25 (37.56)	110.23 (34.74)	0.65
Metabolic syndrome (%)	No	1,265 (60.0)	1,094 (61.5)	1,034 (60.5)	1,088 (61.5)	889 (62.8)	0.515
	Yes	843 (40.0)	686 (38.5)	674 (39.5)	681 (38.5)	526 (37.2)	

Family PIR, family poverty income ratio.

### 3.2 Association between DASH and MetS

The results of the univariable logistic regression analysis of DASH and MetS prevalence can be seen in Supplementary Table S1. Table 2 shows the results of the multivariable logistic regression analysis of DASH and MetS prevalence. The association between DASH diet score (continuous variable) and MetS was significant in our minimally adjusted model (Model 1) (OR = 0.93; 95% CI: 0.91–0.96; *p* < 0.001) and the fully adjusted model (Model 2) (OR = 0.95; 95% CI: 0.92–0.97; *p* < 0.001). This suggests that a 1-unit increase in DASH score was associated with a 5% lower risk of MetS in Model 2.

**Table 2:** Association between DASH and MetS.

DASH quintile		Crude mode l		Model 1		Model 2	
	Cases/participants	OR (95%CI)	*P*	OR (95%CI)	*P*	OR (95%CI)	*P*
DASH	3,410/8,780	0.98 (0.96, 1.0)	0.076	0.93 (0.91, 0.96)	<0.001	0.95 (0.92, 0.97)	<0.001
Quintile1	843/2,108	1.00 (Ref.)		1.00 (Ref.)		1.00 (Ref.)	
Quintile2	686/1,094	0.92 (0.75, 1.12)	0.405	0.80 (0.65, 0.98)	0.034	0.82 (0.66, 1.02)	0.080
Quintile3	674/1,780	0.89 (0.71, 1.11)	0.305	0.71 (0.56, 0.90)	0.006	0.75 (0.58, 0.96)	0.028
Quintile4	681/1,769	0.97 (0.80, 1.18)	0.768	0.73 (0.60, 0.88)	0.001	0.77 (0.63, 0.95)	0.015
Quintile5	526/1,415	0.80 (0.64, 0.99)	0.044	0.54 (0.43, 0.67)	<0.001	0.60 (0.48, 0.77)	<0.001
P-trend		0.138		<0.001		<0.001	

Crude Model: There are no covariates were adjusted.

Model 1: Age and Gender were adjusted.

Model 2: Age, Gender, Race, education level, family poverty–income ratio (PIR), smoking status, and physical activity were adjusted.

In addition, we performed a sensitivity analysis by converting DASH score from a continuous variable to a categorical variable (P0-P25, P25-P50, P50-P75, and P75-P100). In Model 2, participants in the highest DASH score quintile (Q5) had a 40% lower risk of developing MetS compared to the lowest DASH score quintile (Q1), which was statistically significant (OR = 0.60; 95% CI: 0.48–0.77; *p* < 0.001). Compared to the Q 1 group, participants in the Q 2, Q 3, and Q 4 groups also had a 18% (OR = 0.82; 95% CI: 0.66–1.02; *p* = 0.080), 25% (OR = 0.75; 95% CI: 0.58–0.96; *p* = 0.028) and 27% (OR = 0.77; 95% CI: 0.63–0.95; *p* < 0.015) lower risk of developing MetS, and all were statistically significant.

### 3.3 Subgroup analysis

**Figure 2:**
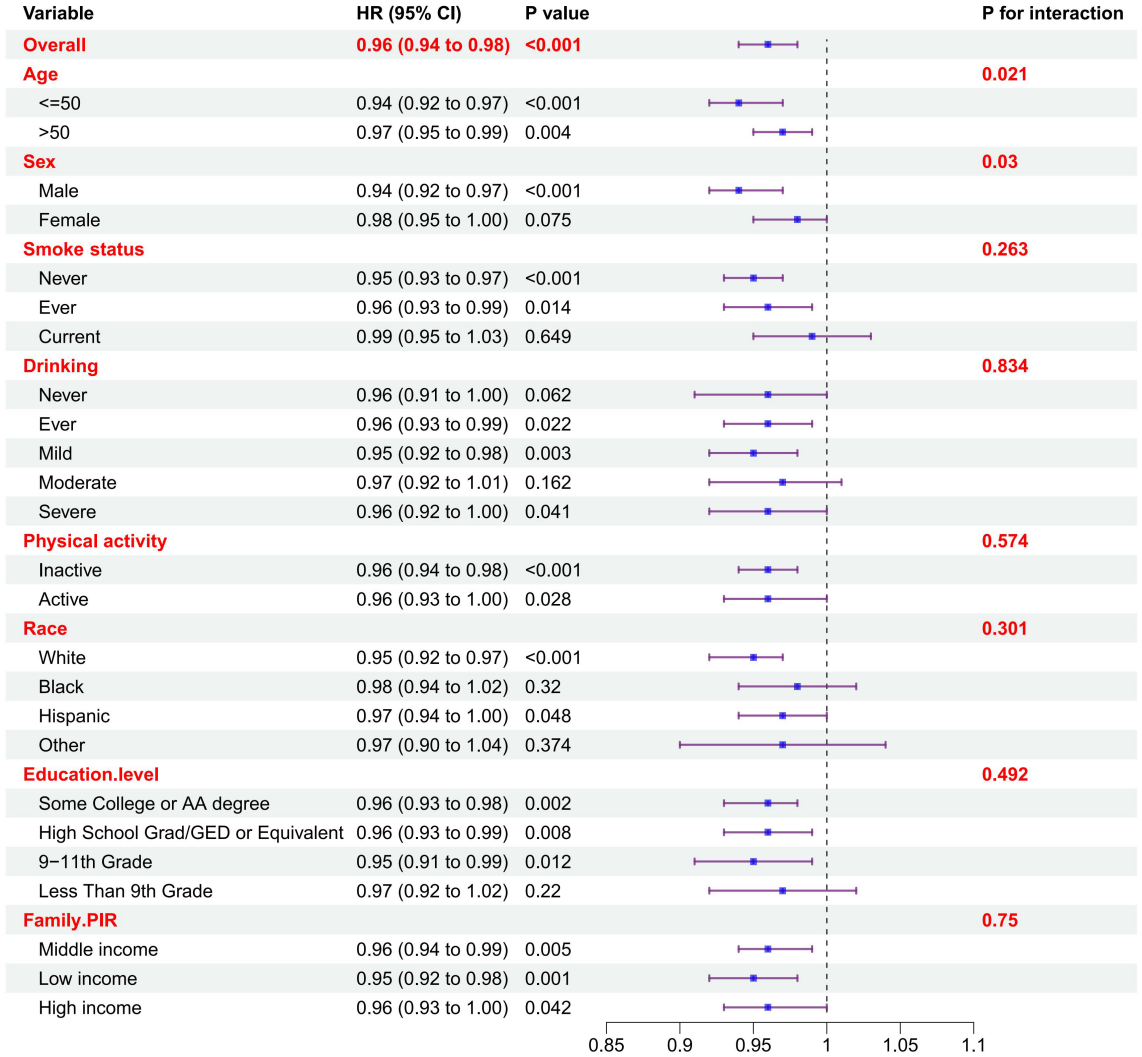
Subgroup analysis of the association between DASH and MetS. Odds ratios were calculated based on DASH scores increased by 1. Each stratum was adjusted for age, gender, race, education level, family income ratio (PIR), smoking status and physical activity.

To further validate the reliability of our results in the presence of confounding variables and to investigate potential factors influencing the association between DASH and the prevalence of metabolic syndrome, we performed subgroup analyses by stratifying key covariates, including age, sex, smoking status, alcohol consumption, race, education level, and family PIR (Figure 2). After adjusting for confounders in the fully adjusted model, statistically significant interactions were observed between age and DASH, sex and DASH (*p* values for interaction <0.05). Meanwhile, the participants who were female, current smokers, and those who consumed alcohol moderately or severely, as well as those black races, people with an education level less than 9th grade, did not exhibit any statistically significant associations between DASH and MetS. Although not statistically significant (*p* > 0.05), a negative correlation between DASH and MetS was observed in the above participants. Compared with the participants, those who were male, younger than 50 years, never smoked, drank little, were white, had a low familial PIR, and those with an education of 9–11 years and a low familial PIR had a lower OR, suggesting that they had a lower risk of MetS. Although education level, familial PIR and race showed trends across quintiles of DASH score, interaction tests showed that the negative correlation between DASH and metabolic syndrome was not influenced by smoking, alcohol consumption, physical activity, race, familial PIR and education level (all *p* > 0.05). However, the interaction between sex and age and DASH appeared to influence the risk of developing metabolic syndrome (interaction *p* < 0.05).

### 3.4 Analysis of restricted cubic spline regression

**Figure 3:**
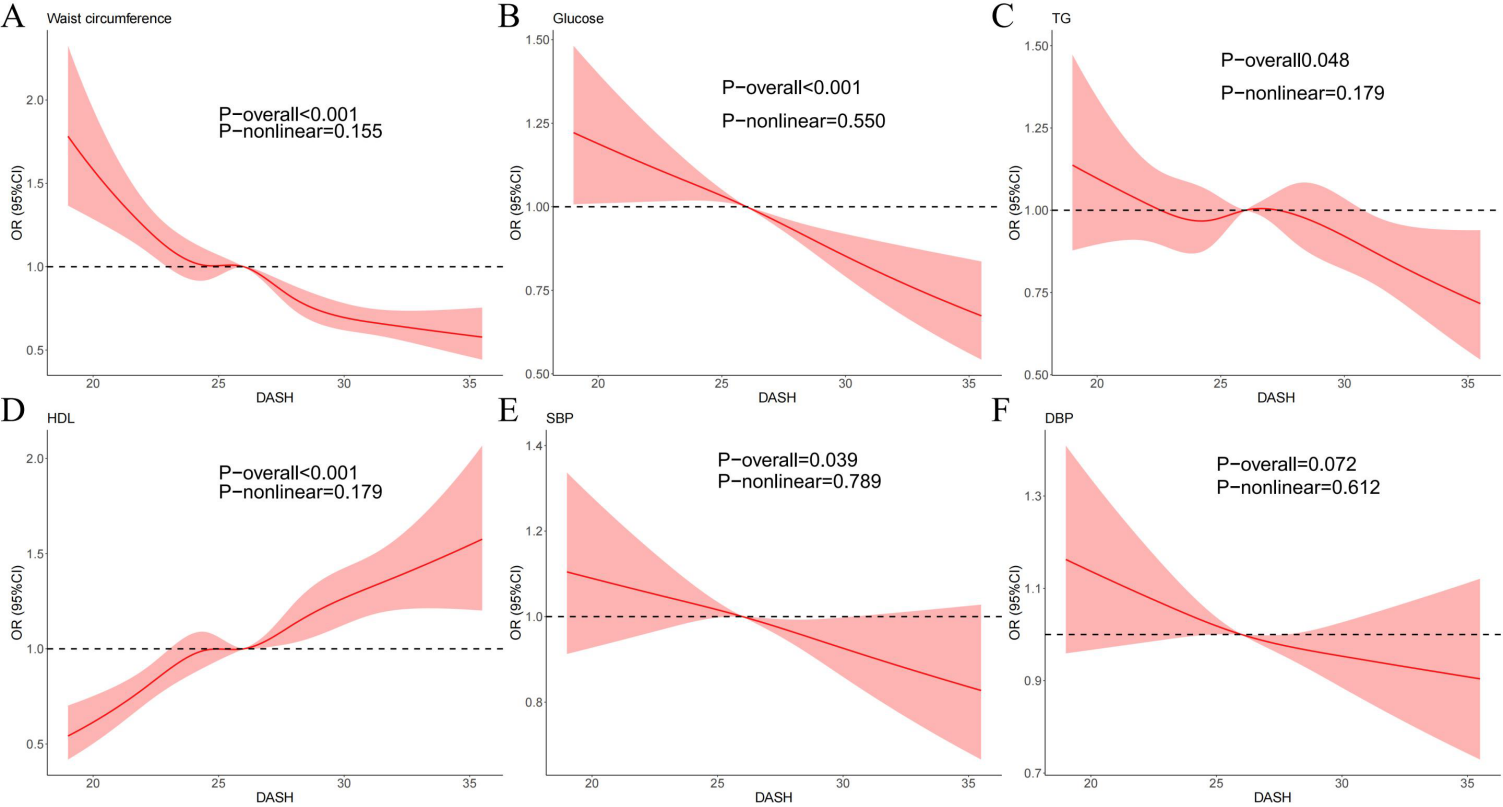
Dose–response relationships between WC **(A)**, FPG **(B)**, TG **(C)**, HDL **(D)**, SBP **(E)**, DBP **(F)**, and DASH. OR, odds ratio; CI, confidence interval. ORs (solid lines) and 95% confidence levels (shaded areas) were adjusted for age, sex, race, education levels, poverty income ratio (PIR), diabetes status, smoking status and physical activity. Vertical dotted lines show the minimum threshold for the positive association with estimated OR = 1.

The relationship between DASH score and various components of metabolic syndrome was shown in Figure 3, including WC, FPG, TG, HDL, SBP, and DBP in the RCS regression. After adjustment for all covariates in model 2 of the primary analysis, a negative linear correlation was observed between DASH score and WC, FPG, TG, DBP, and SBP (P overall < 0.05, P non-linear > 0.05). Conversely, a significant positive linear correlation was observed between DASH score and HDL (P overall < 0.001, P non-linear = 0.179). The linear regression model was used to examine the linear relationship between DASH and the five components of metabolic syndrome (Supplementary Table S2). After adjusting for all covariates, DASH was found to be significantly positively correlated with HDL and negatively correlated with WC, FPG, DBP and SBP.

## 4 Discussion

In this nationally representative sample of US adults, we found that better diet quality, as measured by the DASH diet, was associated with a lower risk of MetS. This association remained significant after adjusting for demographic factors, education, physical activity and smoking. This cross-sectional study was a first in evaluating the impact of the DASH diet on the prevalence of MetS in the general US population. Our findings are significant because MetS has been associated with long-term morbidity and mortality (26, 27).

In recent decades, numerous studies have looked at the DASH diet and demonstrated its positive effects not only on blood pressure but also on various characteristics of the MetS in different population groups (28, 29). In most observational studies, the benefits of the DASH diet were substantial. In addition, the beneficial effects of the DASH diet in lowering certain metabolic traits such as hypertension and dyslipidemia have been well established in clinical trials. Several studies in other populations have provided convincing evidence that greater adherence to the DASH diet can significantly reduce the risk of MetS (30–35) and its components, including central obesity (36) and dyslipidemia.

In addition, a study of 6,826 postmenopausal Korean women aged 49–70 years found that the DASH diet was associated with a lower prevalence of MetS. Kang (33). In their randomized controlled trial in adolescents, Saneei et al. found that adherence to the DASH diet was able to prevent an increase in DBP without affecting SBP levels compared to general dietary recommendations (28). Interestingly, in this broader American population, we found that the DASH diet is associated with a decrease in systolic and diastolic blood pressure (although not significant). However, many studies found that DASH diet was associated with a lower incidence of SBP and DBP (35, 37). In our study, a weighted logistic regression was used for a cross-sectional study. In contrast, previous prospective cohort studies were able to examine temporal associations in more detail and may have captured associations that could be missed in a cross-sectional study such as ours. In our study, DASH data was measured using 24-h dietary recalls, and blood pressure was measured four consecutive times, taking the average value. In comparison, other studies may have had wider recruitment channels, larger sample sizes and longer follow-up periods, which would have improved their ability to detect more subtle associations.

In our study, we also found that men are more sensitive to the DASH diet than women. However, many studies found that greater proportions of participants who were women were in the highest DASH diet score category (38, 39). This may because that our participants consisted primarily of the adult population in the United States, whereas other studies may have focused on other subgroups. The genetic background, lifestyle factors, and environmental influences of our participants might also differ from those in other studies, all of which could affect the relationships between the variables. And the association between the DASH diet and BP levels can be explained by the low sodium content of the DASH diet, which decreases BP in this group. Correspondingly, the meta-analysis performed by He et al. (40) demonstrated that dietary sodium intake is a modifiable risk factor for hypertension at any stage of life. Furthermore, lower sodium intake may lead to improved BP levels through different mechanisms (41). The MASALA study is an ongoing community-based cohort study of South Asian Americans aged 40–84 years, free from cardiovascular disease at enrollment, who reside in the San Francisco or Chicago metropolitan areas (39). And samples of Atena Mahdavi’s study were adolescents with hemophilia from Omid Hospital, Isfahan, Iran (38). Furthermore, no significant association was found between the DASH diet and MetS prevalence in the group of current smokers. In contrast, a high DASH diet was associated with a 0.95 (0.93–0.97) lower risk of MetS than a low DASH diet in the non-smoking group, suggesting that non-smokers responded better to the DASH diet. In the review of randomized clinical trials, Cormick et al. (42) indicated that abundant calcium intake slightly reduced systolic BP and diastolic BP, especially in normotensive young adults. On the other hand, a higher intake of fruits, vegetables, and legumes, together with less SFA and more monounsaturated fatty acid consumption, may explain the beneficial effects of the DASH diet on the parameters of abdominal fat and waist circumference. A randomized controlled study (43) of people with type 2 diabetes confirmed that after the DASH diet, the mean change for HDL cholesterol levels was higher (4.3 ± 0.9 mg/dL; *p* = 0.001) and LDL cholesterol was reduced (−17.2 ± 3.5 mg/dL; *p* = 0.02). It may be because DASH diet helps to improve insulin sensitivity. Insulin resistance is closely related to dyslipidemia. When insulin sensitivity is improved, it can better regulate lipid metabolism and promote the synthesis and secretion of HDL, thereby increasing the concentration of HDL in the blood.

Furthermore, subgroup analysis showed no significant interaction between the DASH diet and metabolic syndrome in individuals with different drinking status, physical activity, education level, family PIR and race. However, among the five quintiles of the DASH diet, ethnicity, household income ratio and education level all showed a regular distribution. For example, the proportion of Hispanics gradually increased from the lowest to the highest DASH quintile, while the proportion of other ethnic groups gradually decreased. The possible reason for this could be that the Hispanic culture places great importance on family and nutrition (44). They are more likely to accept and practice healthy eating habits. Also, Hispanic cuisine consists mainly of fresh fruits and vegetables, seafood and olive oil, all of which help prevent hypertension. In terms of household income, low-income families were less likely to adhere to the DASH diet than high-income families. This was because people with higher incomes lived in areas that were rich in food, less sensitive to food prices and had the support of professional health services, so they were better able to implement the DASH diet and thus reduce the risk of cardiovascular disease. In contrast, people on low incomes often faced the dilemma of “food deserts.” They often chose low-cost but nutrient-poor foods, had no support from professional services, and had difficulty adhering to the DASH diet, which affected the improvement of cardiovascular indicators (45). The proportion of people with a high level of education was the highest, gradually increasing from the lowest DASH quintile to the highest. The possible reason for this was that people with a higher level of education were generally better able to acquire and understand information. They were able to understand and strictly adhere to the benefits of the DASH diet. People with a lower level of education, on the other hand, may have difficulty fully grasping the importance of the DASH diet due to a lack of knowledge, resulting in a low practical effect (46).

However, in their cohort study of 6,851 Spanish participants over a period of 8.3 years, Pimenta et al. investigated the association between adherence to the DASH diet and the risk of MetS. In the final model, adherence to the DASH diet was not associated with the risk of MetS after adjusting for relevant variables [RR = 0.82; 95% CI: 0.64–1.03; *p* = 0.083] (47). In contrast, in our study, in which participants were categorized into DASH quintiles, the OR of MetS risk was only 0.60 (0.51–0.71) in the highest DASH quintile (Q5) compared to the lowest quintile (Q1).

The DASH diet may influence MetS risk via different biological mechanisms (41). High dietary fiber has been reported to improve indicators of metabolic abnormalities such as FPG, TG, blood pressure, and WC by reducing appetite, dietary energy density, and total energy intake (48). The higher intake of fiber, folate, potassium, magnesium, calcium, vitamin C and phytochemicals such as plant sterols, carotenoids and flavonoids in the DASH diet may have a protective effect on blood pressure, lipid profiles, insulin sensitivity, total antioxidant capacity and reduced risk of MetS (49). Dietary fiber ferments in the intestine and forms short-chain fatty acids, which improve the intestinal barrier function and can promote the absorption of potassium and magnesium. Potassium and magnesium act synergistically on the ion channels in the cell membrane to regulate cell excitability and vascular tone, thus reducing the risk of MetS.

Another mechanism by which the DASH diet reduces the risk of MetS is the higher intake of low-fat dairy products. Calcium influences insulin sensitivity, blood pressure and lipid concentrations (49). Calcium is negatively correlated with levels of the above cardiometabolic factors via regulation of calciotropic hormones, increased excretion of bile acid and fecal fat, decreased levels of intracellular calcium, and influence on the metabolism of other electrolytes such as sodium. In addition, it has been shown that not only adherence to the DASH diet and limiting sodium intake, but also consumption of potassium-rich foods can improve blood pressure and reduce hypertension (41).

This study has some limitations. The NHANES study is a cross-sectional survey and the results do not allow conclusions to be drawn about direction or causality. Furthermore, in our study, DASH scores were classified on the basis of percentages. However, there were significant differences in the DASH score ranges represented by each category. For example, the DASH score for the fifth category was 29–37, while the scores for the first, second, third, and fourth categories were 18–24, 24–25.5, 25.5–27, and 27–29, respectively. For the narrower and more similar middle categories, these intervals were relatively concentrated, suggesting that the majority of the population adhered to the DASH diet at a moderate level. Furthermore, each additional point in the DASH score might correspond to small but significant changes in components of metabolic syndrome. The broader fifth category comprised a range from a relatively high to an extremely high degree of DASH adherence, suggesting that the dietary habits of individuals within this range were already highly compliant with the DASH dietary requirements, although there were still individual differences. On the one hand, researchers need to further subdivide this interval to investigate whether there are subtle differences in the impact of different levels of DASH adherence within the high-score segment on metabolic syndrome. On the other hand, this wide interval also suggests that when recruiting for clinical research, if the research goal is to explore the ultimate benefits of the DASH diet, more rigorous screening is needed to ensure that only individuals with a truly extremely high level of adherence are included, rather than treating all individuals in this interval as a homogeneous group to avoid outcome bias. Although potential confounders were adjusted for in this study, the possibility of remaining confounders could not be completely ruled out, in this case, the magnitude of change in each indicator corresponding to a unit change in DASH score may be overestimated or underestimated. Our study focused on the American population, which limits its generalizability, For populations in other regions, the degree of change in individual indicators (such as TG, WC, etc.) may vary when the DASH score increases by 1 unit. Despite these limitations, the external validity of the results was enhanced by the nationally representative sample, the breadth of variables examined in this study, and the range of sociodemographic diversity in the study population. Our study confirms the higher adherence to the DASH diet is inversely associated with the risk of MetS in the US population. Interventions to improve diet quality should be a public health priority.

## 5 Conclusion

Our findings supported the hypothesis that DASH levels were inversely associated with MetS. Furthermore, this association was noted in all ethnic groups examined. Moreover, this study treated DASH as a categorical variable (i.e., the DASH quintile) for ease of interpretation. Future large intervention studies and well-designed observational studies may provide more evidence on whether improving DASH levels can reduce the incidence of MetS.

## Data Availability

The datasets presented in this study can be found in online repositories. The names of the repository/repositories and accession number(s) can be found below: https://www.cdc.gov/nchs/nhanes/index.htm.
